# 

**Published:** 1892-12-03

**Authors:** 


### CONTENTS.

| | PAGE |
|---|---|
| **Cremation or Burial?** | 145 |
| **Passing Topics.**—The Sunday Fund Meeting | 146 |
| **Medical History from the Earliest Times.** By E. A. Withington, M.A., M.B.Oxon. XXXIV.—The Darkest Age | 147 |
| **The Attitude of Great Writers Towards Disease.** XVII. —Charles Kingsley | 148 |
| **Diet in Disease.** By Mrs. Ernest Hart. VIII.—Digestion | 149 |
| **Everybody's Page** | 150 |
| **Annotations.**—A Vote of Confidence for Modern Medicine—Four Years of Margarine—Trained Nurses and Private Patients | 151 |
| **Notes and News** | 152 |
| **Books Received** | 156 |
| **Editor's Letter Box**—Anti-Vivisectionists and their Mistakes | 160 |
| **The Student of Medicine**—Notes from the Schools, &c. | |
| **The Hospital Clinic—** | |
| Paddington Green Children's Hospital.—The Treatment of Spinal Disease | 153 |
| Central London Hospital.—Treatment of Inflammation of the Middle Ear | 154 |
| St. Mary's Hospital.—The Treatment of Enteric Fever | 155 |
| **The Institutional Workshop—** | |
| Within the Hospitals.—Bolingbroke House Pay Hospital | 157 |
| Hospital Opinion.—The Hospital for Curing the Poor in Their Own Homes of Loss of Movement from Paralysis, Rheumatism, and Deformity | 158 |
| Practical Departments.—Bedsteads I. | 158 |
| Colonial Institutions.—Asylums of New Zealand | 159 |
| Hospital Meetings.—The Middlesex Hospital | 160 |

### EXTRA SUPPLEMENT.—THE NURSING MIRROR.

| | |
|---|---|
| **En Passant.**—Lady Doctors—The Trained Nurses' Club—An International Nursing Congress—Festivities in the Emerald Isle —Private Nurses' Association and Club, Dublin—Short Items—Jottings from India | lxi |
| **The Registration of Nurses and the R.B.N.A.**—Application to the Privy Council | lxii |
| **Notes and Queries** | lxxxi |

